# Adenoid Cystic Carcinoma of the Breast: Clinicopathological Features and Long-Term Outcomes from a 20-Year Retrospective Cohort

**DOI:** 10.32604/or.2026.085356

**Published:** 2026-08-13

**Authors:** Iseult M. Browne, Susanna Slater, Myrto Kastrisiou, Mae Alghawas, Edward Phillips, Mark Beresford, Stephen R. D. Johnston, Zoe Kemp, Emma Kipps, Marina Parton, Nicholas C. Turner, Alicia F. C. Okines

**Affiliations:** 1 The Breast Unit, The Royal Marsden NHS Foundation Trust, London, UK; 2 The Breast Cancer Now Toby Robins Research Centre, The Institute of Cancer Research, London, UK; 3 Royal United Hospital Bath, Somerset, UK

**Keywords:** Adenoid cystic carcinoma, breast cancer, triple-negative breast cancer, retrospective study, locoregional therapy, rare breast tumour

## Abstract

Background: Adenoid cystic carcinoma (ACC) of the breast is a rare triple-negative malignancy with an indolent clinical course distinct from conventional triple-negative breast cancer (TNBC). Optimal management remains undefined due to limited prospective data. This study aimed to characterise the clinicopathological features, treatment patterns, and long-term outcomes of breast ACC at a high-volume specialist centre, contributing real-world evidence to inform management in the absence of prospective trial data. Methods: A single-institution retrospective cohort study was conducted of 24 patients with histopathologically confirmed breast ACC treated at The Royal Marsden NHS Foundation Trust between 2000 and 2020. Clinicopathological and outcome data were analysed descriptively; overall survival (OS), disease-specific survival (DSS), and relapse-free survival (RFS) were estimated using the Kaplan–Meier method. Results: Median age was 57 years. Nodal involvement was rare (8%). Adjuvant radiotherapy was administered in 88% of patients; only two patients (8%) received chemotherapy for breast ACC. Five patients (21%) experienced disease relapse after a median of 2.3 years (range 1.3–14.0). The estimated 5- and 10-year OS were both 88.4% (95% CI 74.5–100%) and DSS were both 93.3% (95% CI 81.5–100%). Conclusions: To our knowledge this represents the largest single-institution cohort study of breast ACC reported to date. The clinical behaviour of breast ACC more closely resembles salivary gland ACC than conventional TNBC, supporting a conservative locoregionally focused management approach and questioning the routine use of chemotherapy on the basis of triple-negative receptor status alone. The propensity for late relapse supports long-term surveillance beyond the standard 5-year window.

## 1 Introduction

Adenoid cystic carcinoma (ACC) is a rare malignancy most commonly arising in the major and minor salivary glands, but also occurring at other sites including the breast, lung, and skin [1]. Breast ACC accounts for approximately 0.1–1% of all breast malignancies, rendering it one of the rarest histological subtypes in clinical practice [2,3]. Despite this rarity, breast ACC carries significant clinical importance due to its paradoxical immunophenotype. It is consistently triple negative, lacking expression of oestrogen receptor (ER), progesterone receptor (PR), and lacking human epidermal growth factor receptor 2 (HER2) overexpression or amplification [4,5]. Conventional triple-negative breast cancer (TNBC) is associated with an aggressive clinical course, high rates of distant metastasis, and poor prognosis [6]. In contrast, breast ACC behaves in a manner most similar to salivary gland ACC, characterised by low histological grade, low Ki-67 proliferative index, infrequent lymph node involvement, and a highly favourable long-term prognosis with 5- and 10-year overall survival (OS) rates reported between 85% and 100% [3,7,8]. This biological divergence has important therapeutic implications: the reflexive application of TNBC chemotherapy protocols to breast ACC may risk overtreatment in a disease predominantly managed with locoregional therapies, and underscores the need for accurate histological subtype recognition in clinical practice.

From a histopathological perspective, breast ACC demonstrates a biphasic morphology consisting of luminal epithelial and myoepithelial cells arranged in cribriform, tubular, or solid patterns, closely mirroring its salivary gland counterpart [3]. The current World Health Organisation (WHO) classification of breast tumours recognises three principal subtypes: classic ACC (Classic), solid-basaloid ACC (SB-ACC), and ACC with high-grade transformation (HGT-ACC) [9]. Classic ACC is the predominant variant, characterised by well-formed cribriform and tubular structures with variable solid architecture, a low mitotic rate, and pushing tumour margins, and is associated with an indolent clinical course and excellent long-term prognosis [10]. Solid-basaloid ACC, by contrast, is defined by predominantly solid growth composed of basaloid cells, with higher nuclear grade, increased mitotic activity, frequent necrosis, and scant cytoplasm, and is associated with a higher rate of local recurrence and, rarely, distant metastasis [11]. ACC with high-grade transformation is an exceptionally rare variant characterised by the presence of a conventional ACC component alongside a morphologically distinct high-grade carcinoma component that has lost the biphasic differentiation characteristic of ACC, analogous to dedifferentiation described in salivary gland ACC, and is associated with an aggressive clinical course and poor prognosis [12]. Accurate subtype classification is therefore clinically meaningful and should be routinely performed by specialist breast pathologists. Immunohistochemically, ACC retains expression of basal/myoepithelial markers such as p63 and cytokeratin 5/6, further distinguishing it from conventional TNBC [13].

The molecular basis of ACC has been increasingly elucidated over the past decade. Breast ACC is characterised by a recurrent chromosomal translocation t(6;9)(q22–23;p23–24), resulting in fusion of the *MYB* and *NFIB* transcription factor genes. The MYB-NFIB fusion protein acts as a constitutive transcriptional activator, driving oncogenic gene expression programmes including upregulation of proliferative, anti-apoptotic, and stemness-related pathways. This fusion is a frequent molecular event in ACC, detected in a significant proportion of cases across all anatomical sites and serves as a diagnostically and biologically defining feature of the tumour [14,15,16,17].

Beyond the *MYB-NFIB* fusion, recurrent molecular events in breast ACC include mutations affecting chromatin remodelling genes [18,19], with *NOTCH1* and *NOTCH2* signalling component mutations additionally reported and particularly enriched in the SB-ACC subtype [18]. Breast ACC exhibits a strikingly low somatic mutational burden [19], in sharp contrast to basal-like TNBC which is typically characterised by widespread genomic instability, *TP53* mutations, and complex copy number landscapes. This genomic simplicity is consistent with the indolent clinical behaviour of breast ACC and further distinguishes it at a molecular level from conventional TNBC. While *BRCA*1/2 mutations are enriched in basal-like TNBC relative to other breast cancer subtypes, they are not typically associated with breast ACC [19]. This molecular signature differentiates breast ACC from other TNBC subtypes and likely underlies its limited sensitivity to conventional cytotoxic chemotherapy.

Emerging molecularly targeted strategies are under active investigation for ACC across anatomical sites [20]. MYB pathway inhibition represents a promising preclinical therapeutic strategy, with compounds demonstrating activity in ACC cell lines and patient-derived xenograft models [21]. Additional oncogenic pathways, including c-KIT, EGFR, and NOTCH1, have been implicated in tumorigenesis and represent potential therapeutic targets [20]. FGFR pathway dysregulation has been identified in a subset of ACC tumours, providing a biological rationale for FGFR-directed therapy. Early-phase clinical studies of multi-kinase inhibitors with FGFR activity, including dovitinib and lenvatinib [22,23], have demonstrated modest partial responses and disease stabilisation in advanced ACC. Isolated case reports further suggest that selective FGFR inhibition, for example, futibatinib in a tumour harbouring an *FGFR2* alteration, may confer meaningful clinical benefit in molecularly selected patients [24], though prospective validation in larger cohorts is needed.

Optimal management of breast ACC remains undefined. Existing evidence is largely limited to case reports, small single-centre series, and population-based registry analyses, each with inherent limitations: registry data are constrained by inconsistent pathological classification over time, absence of WHO subtype information, and limited treatment detail; small series lack statistical power; and no prospective randomised data exist or are feasible given the rarity of this tumour. As a result, management of breast ACC is largely extrapolated from paradigms for salivary gland ACC and conventional TNBC, with a risk of both undertreatment and overtreatment. Key outstanding questions include the role of sentinel lymph node biopsy versus axillary lymph node dissection, optimal surgical margins, the utility of adjuvant radiotherapy following breast-conserving surgery, and the indications for systemic therapy in both early and metastatic settings.

Analyses of the Surveillance, Epidemiology, and End Results (SEER) database have identified several hundred cases accrued over decades, confirming the predominantly post-menopausal female demographic, the rarity of nodal involvement, and favourable survival outcomes of breast ACC [25]. Population-level data have also informed adjuvant treatment decision-making in this subtype [26]. However, registry data are limited by inconsistent pathological classification over time, absence of detailed treatment information, and the inability to distinguish between WHO subtypes. Single-institution series, while numerically smaller, offer substantially richer clinicopathological and outcome data and reflect the evolving management approaches of specialist centres.

Several critical knowledge gaps remain unaddressed in the published literature. WHO subtype-specific outcome data are largely lacking. Long-term follow-up data extending beyond 10 years are sparse, despite the well-recognised propensity for late relapse in breast ACC occurring years or even decades after diagnosis [27]. Detailed treatment and outcome data from high-volume specialist centres reflecting contemporary multidisciplinary management are absent from the published evidence base.

The Royal Marsden NHS Foundation Trust (London, UK) is a specialist cancer centre with a long-standing track record in the diagnosis and management of rare breast tumours. Here we present a 20-year single-institution, multi-site, retrospective analysis of all patients with histopathologically confirmed breast ACC treated at The Royal Marsden NHS Foundation Trust between 2000 and 2020. The primary aim of this study was to characterise the clinicopathological features, treatment patterns, and long-term outcomes of breast ACC in a specialist National Health Service centre, with the objective of contributing real-world evidence to inform management decision-making in the absence of prospective trial data. This series represents, to our knowledge, the largest single-institution cohort of breast ACC reported to date. Existing published series have been limited by small sample sizes, incomplete pathological subtyping, short follow-up duration, and absence of detailed treatment data. This study addresses these gaps by providing detailed clinicopathological characterisation including WHO subtype distribution, Kaplan-Meier estimates of OS, DSS, and RFS with extended follow-up beyond 20 years, and real-world management data from a high-volume specialist National Health Service centre, contributing to the limited evidence base available for this rare malignancy in the absence of prospective trial data.

## 2 Materials and Methods

### 2.1 Study Design and Setting

We conducted a single-institution multi-site retrospective cohort study over a 20-year period (January 2000 to December 2020) across three hospital sites in London: The Royal Marsden NHS Foundation Trust (Chelsea, Sutton and Kingston sites). The study aimed to characterise the clinicopathological features and long-term outcomes of patients with ACC of the breast treated within this network, reflecting real-world specialist centre practice. Patients were identified through a search of the institutional electronic health records (EHR) system. All pathology specimens were reviewed by specialist breast pathologists and diagnosis was confirmed according to the World Health Organisation (WHO) classification of Tumours of the Breast, incorporating both morphological assessment and immunohistochemical staining to assign histological subtype. Where clinically indicated, central pathology re-review of archival slides was performed at the discretion of the treating multidisciplinary team. The study was approved by the institutional review board at The Royal Marsden NHS Foundation Trust (Protocol Code: SE1028). Given the retrospective nature of the study and use of anonymised patient data, informed consent was waived in accordance with UK Health Research Authority guidance.

### 2.2 Eligibility Criteria

Eligible patients had a histopathologically confirmed diagnosis of ACC of the breast and received any component of management (surgery, radiotherapy, or systemic therapy) at The Royal Marsden NHS Foundation Trust between January 2000 and December 2020, with clinicopathological data available within the institutional EHR system. Patients were excluded if a formal histopathological diagnosis of primary breast ACC was not confirmed within the institutional electronic health records, or if insufficient clinicopathological data were available to contribute meaningfully to the descriptive analysis. Patients with incomplete demographic, treatment, or outcome data were retained in analyses where possible; and the proportion of missing data for each variable is reported in relevant tables.

### 2.3 Data Collection

Electronic patient records were reviewed to extract the following variables: demographic and clinical characteristics including age at diagnosis, sex, and menopausal status; pathological features including tumour size, histological subtype, disease focality (unifocal or multifocal), tumour grade, surgical margin status, lymphovascular invasion (LVI), Ki-67 proliferative index, and ER, PR, and HER2 status; axillary nodal status; and treatment details including type of breast and axillary surgery, neoadjuvant and adjuvant radiotherapy, systemic chemotherapy, and any therapy administered for relapsed disease. Outcome data included time to relapse, site(s) of relapse, overall survival, disease-specific survival, relapse-free survival, and cause of death.

Follow-up data were obtained from electronic health records including outpatient clinic letters, imaging reports, multidisciplinary team meeting records, and correspondence with referring teams. Surveillance protocols varied across the 20-year study period and between treating clinicians, reflecting real-world specialist centre practice.

### 2.4 Statistical Analysis

Descriptive statistics were used to summarise baseline characteristics and treatment data. Continuous variables were reported as median and range; categorical variables as frequencies and percentages. Median follow-up was calculated from the date of histological diagnosis to the date of last known follow-up or death. Overall survival (OS) was defined as the time from date of histological diagnosis to death from any cause or last follow-up. Disease-specific survival (DSS) was defined as the time from date of histological diagnosis to death attributable to ACC; patients who died from unrelated causes were censored at their date of death. Relapse-free survival (RFS) was defined as the time from date of histological diagnosis to first documented disease relapse or death from any cause, whichever occurred first; patients without a relapse event were censored at the date of last follow-up. Patients lost to follow-up were censored at the date of last recorded clinical contact. OS, DSS, and RFS were estimated using the Kaplan–Meier method. All statistical analyses were performed using R Statistical Software (version 4.3.1; R Foundation for Statistical Computing, Vienna, Austria).

## 3 Results

### 3.1 Patient Demographics and Baseline Characteristics

Twenty-four patients with histopathologically confirmed breast ACC were identified over the 20-year study period. All patients were female. Median age at diagnosis was 57 years (range: 36–82 years). Median follow-up from date of histological diagnosis was 58.6 months (range 2.7–273.5 months). Seventeen patients (70.8%) were post-menopausal at diagnosis. All 24 patients were ER-negative and PR-negative. No patients were HER2 positive, two patients were HER2 1+.

### 3.2 Clinicopathological Features

Clinicopathological features are summarised in Table 1. The predominant WHO histological subtype was classic ACC, identified in 21 patients (88%). Solid-basaloid ACC was present in 2 patients (8%), and one patient (4%) had ACC with high-grade transformation. With regards to tumour stage, T2 disease was most common, identified in 14 patients (58%), followed by T1 in 8 patients (33%), and two patients (8%) presented with T3 disease.

**Table 1:** Baseline demographics and clinicopathological characteristics of patients with breast ACC.

Characteristic	n (%)
**Demographics**	
Total patients	24
Female sex	24 (100%)
Median age at diagnosis, years (range)	57 (36–82)
Multifocal disease	1 (4%)
**Receptor status**	
ER negative	24 (100%)
PR negative	24 (100%)
HER2 0	22 (92%)
HER2 1+	2 (8%)
**Tumour characteristics**	
Median primary tumour size, mm (range)^1^	24 (10–60)
T1 (≤20 mm)	8 (33%)
T2 (>20–50 mm)	14 (58%)
T3 (>50 mm)	2 (8%)
**WHO histological subtype**	
Classic ACC	21 (88%)
Solid-basaloid ACC	2 (8%)
ACC with high-grade transformation	1 (4%)
**Tumour grade**	
Grade 1	4 (17%)
Grade 2	12 (50%)
Grade 3	4 (17%)
Not stated/not assessable	4 (17%)
**Ki-67 proliferative index**	
Not documented	19 (79%)
0–10%	2 (8%)
10–50%	2 (8%)
>50%	1 (4%)
**Lymphovascular invasion**	
Absent	22 (92%)
Unknown	2 (8%)
**Nodal status**	
Node negative	21 (88%)
N1 macrometastasis	1 (4%)
N1 micrometastasis (N1mi)	1 (4%)
Unknown^2^	1 (4%)
**BRCA germline status**	
Wild type	7 (29%)
Not tested/unknown	17 (71%)

All percentages based on n = 24; ^1^Size data available for all 24 patients. One patient received neoadjuvant chemotherapy prior to surgery; the pre-neoadjuvant clinical tumour size (60 mm, cT3) is reported for staging purposes; post-neoadjuvant pathological tumour size was 40 mm. ^2^Nodal status unknown in one patient who did not have surgery. ACC = adenoid cystic carcinoma; ER = oestrogen receptor; HER2 = human epidermal growth factor receptor 2; PR = progesterone receptor; WHO = World Health Organisation.

Tumour grade 2 was seen in 12 patients (50%), with grade 1 in four (17%), grade 3 in four (17%), and grade not stated or assessable in four (17%). LVI was absent in 22 patients (92%) and unknown in two patients (8%). One patient (4%) had multifocal disease at presentation. Clinical nodal status was node-negative in 21 patients (88%), with one patient having macrometastatic nodal disease (4%), one patient having micrometastatic nodal disease (4%) and one patient with unknown nodal status (4%).

*BRCA* germline testing was documented as wild type in seven patients (29%), and testing had not been performed, or results were unavailable in the remaining 17 patients (71%). Ki-67 data were incompletely documented, with no recorded value in 19 patients (79%). Among those with available Ki-67 data, values ranged from 0–10% (two patients, 8%) to 10–50% (two patients, 8%), with one patient (4%) demonstrating Ki-67 greater than 50%.

### 3.3 Surgical Management

Wide local excision (WLE) with either sentinel lymph node biopsy (SLNB) or axillary lymph node dissection (ALND) was performed in 18 (75%) of patients. Of these, four patients (22% of WLE cases) required re-excision of margins to achieve adequate clearance. Close surgical margins were defined as tumour within 1 mm of the inked surgical margin, consistent with standard UK histopathological reporting practice. It is acknowledged that margin reporting conventions evolved over the 20-year study period. Mastectomy was performed in five patients (21%), including one patient who converted from WLE to mastectomy after repeated margin involvement, and one patient who underwent mastectomy following neoadjuvant chemotherapy. One patient did not undergo breast surgery, having received chemotherapy for a synchronous lung cancer. Surgical management data are summarised in Table 2. 

**Table 2:** Surgical management of breast ACC.

Surgical Management	n (%)
**Breast surgery**	
Wide local excision (WLE)	18 (75%)
Re-excision of margins required	4 (22%)^1^
Mastectomy	5 (21%)
Conversion: WLE → mastectomy	1
No breast surgery	1 (4%)
**Axillary surgery**	
Sentinel lymph node biopsy (SLNB)	17 (71%)
Axillary lymph node dissection (ALND)	5 (21%)
One-step nucleic acid amplification (OSNA)	1 (4%)
No axillary surgery	1 (4%)
**Surgical margin status^2^**	
Clear after first excision	14 (61%)
Clear after re-excision	5 (22%)
Close margins (not re-excised)	4 (17%)

^1^Of 18 WLE patients; 4/18 of WLE patients (22%). ^2^Margin status reported for 23 patients (excludes the 1 patient who did not undergo breast surgery).

### 3.4 Adjuvant Therapy

Adjuvant radiotherapy was administered to 21 patients (88%). One patient received neoadjuvant chemotherapy prior to surgery. On presentation this patient had a large inoperable tumour that was 60 mm, grade 3 and involved the skin, with extensive lymph node involvement. They received four cycles of epirubicin and cyclophosphamide followed by a mastectomy and axillary lymph node dissection. This demonstrated a response to neoadjuvant chemotherapy with 40 mm of residual IDC but no residual ACC, and one lymph node macrometastasis. One patient received adjuvant chemotherapy following surgery, after a discussion at the multidisciplinary team meeting (MDT) based on the multi-focality of the tumour. One further patient received chemotherapy for a synchronous advanced lung cancer diagnosis, and did not receive any treatment directed at breast ACC. No patient received targeted therapy or immunotherapy as part of primary treatment. Adjuvant therapy data are summarised in Table 3.

**Table 3:** Adjuvant therapy administered for breast ACC.

Treatment	n (%)
**Adjuvant Radiotherapy**	
Yes	21 (88%)
No^1^	3 (13%)^1^
**Neoadjuvant Chemotherapy**	
Yes (Regimen EC)	1 (4%)
No	23 (96%)
**Adjuvant Systemic Therapy**	
Yes (Regimen: FEC)	1 (4%)
No	23 (96%)
**Any Chemotherapy for Breast ACC**	
Yes^2^	2 (8%)
No	22 (92%)

^1^Radiotherapy not administered in 3 patients: 2 who underwent mastectomy and 1 who did not undergo breast surgery as they were treated for a synchronous lung cancer.^2^One additional patient received chemotherapy for a synchronous lung cancer without breast surgery or radiotherapy; this patient is excluded from breast ACC systemic therapy figures. EC = epirubicin/cyclophosphamide; FEC = fluorouracil, epirubicin, cyclophosphamide.

### 3.5 Relapse and Survival Outcomes

**Figure 1:**
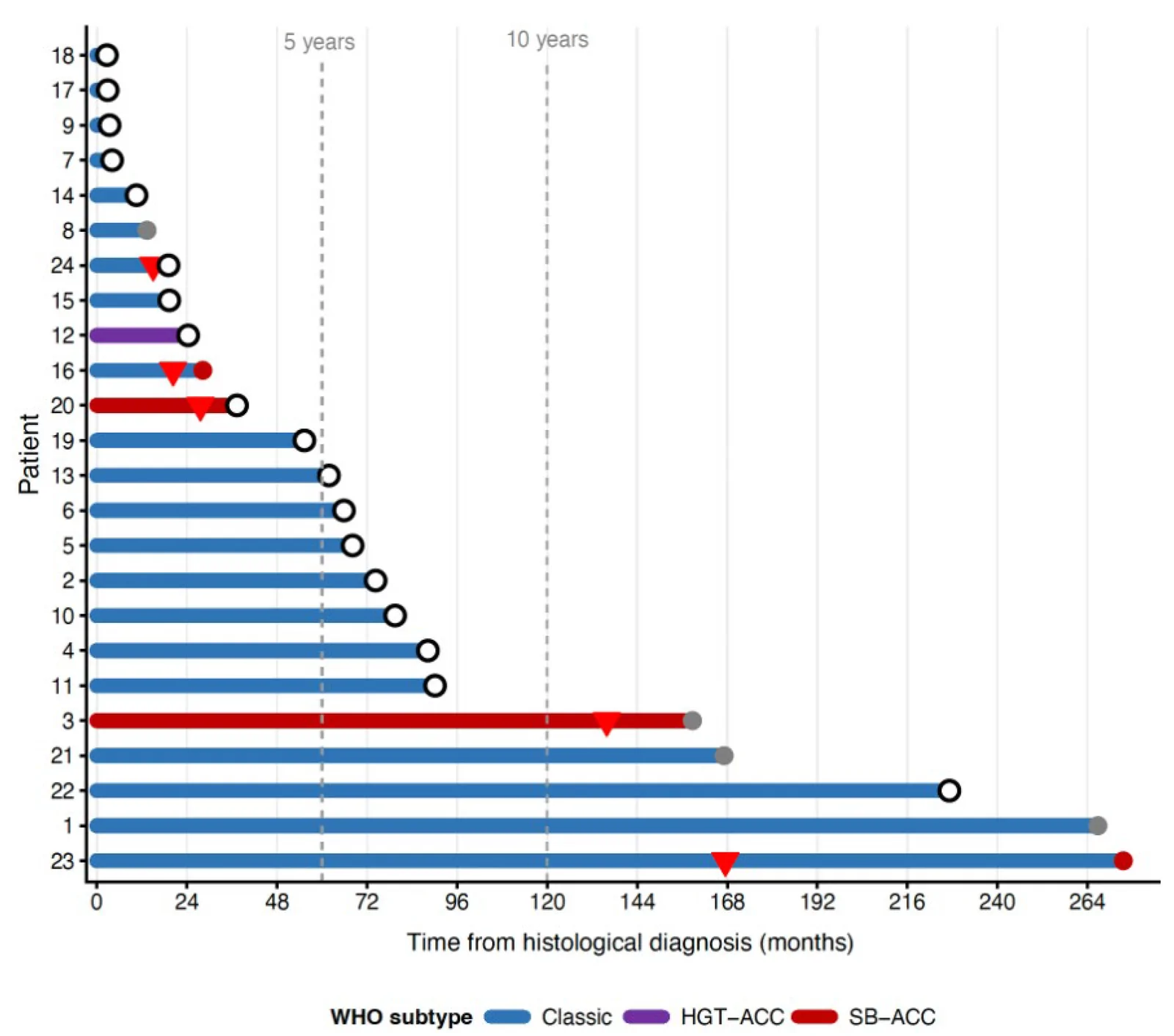
Swimmer plot showing individual patient follow-up, relapse, and survival outcomes for 24 patients with breast ACC. Each horizontal bar represents one patient, with length corresponding to duration of follow-up from date of histological diagnosis. Bar colour indicates WHO histological subtype: blue = classic ACC; red = solid-basaloid ACC (SB-ACC); purple = ACC with high-grade transformation (HGT). Symbols: filled red downward triangle (▼) = disease relapse; open circle (○) = alive at last follow-up; filled red circle (●) = died from ACC; filled grey circle (●) = died from unrelated cause. Dashed vertical lines indicate 5-year and 10-year timepoints. ACC = adenoid cystic carcinoma; HGT = high-grade transformation; SB-ACC = solid-basaloid adenoid cystic carcinoma.

Five patients (21%) experienced disease relapse. The median relapse-free interval was 2.3 years (range: 1.3–14.0 years), highlighting the protracted natural history of this disease; individual patient follow-up and relapse data are illustrated in Fig. 1. Sites of relapse included the ipsilateral breast, chest wall, lung, and bone. Notably, both solid-basaloid ACC patients in the cohort experienced relapse (2/2, 100%), compared with 3 of 22 non-solid-basaloid ACC patients (14%). Four relapsed patients (80%) received active treatment: one with surgery, chemotherapy, and radiotherapy in combination, one with chemotherapy alone, one with radiotherapy alone, and one with surgery alone. One patient did not receive active treatment for their relapse and subsequently died with disease. Two relapses had atypical histology: one patient developed local recurrence with metaplastic carcinoma at the site of prior SB-ACC, suggesting dual pathology rather than true ACC recurrence; and one patient developed local recurrence with carcinoma exhibiting neuroendocrine features following neoadjuvant chemotherapy, most likely representing progression of a concurrent IDC component.

Kaplan–Meier analysis demonstrated favourable survival outcomes. The estimated 5-year and 10-year OS were both 88.4% (95% CI 74.5–100%) and the estimated 5-year and 10-year DSS were both 93.3% (95% CI 81.5–100%), with no deaths occurring between 5 and 10 years of follow-up. The estimated 5-year and 10-year relapse-free survival (RFS) were both 82.2% (95% CI 65.8–100%), with no relapses observed between 5 and 10 years of follow-up. Two further relapses occurred beyond 11 years of follow-up, at 11.3 and 14.0 years respectively, underscoring the propensity for late relapse characteristic of this malignancy.

**Figure 2:**
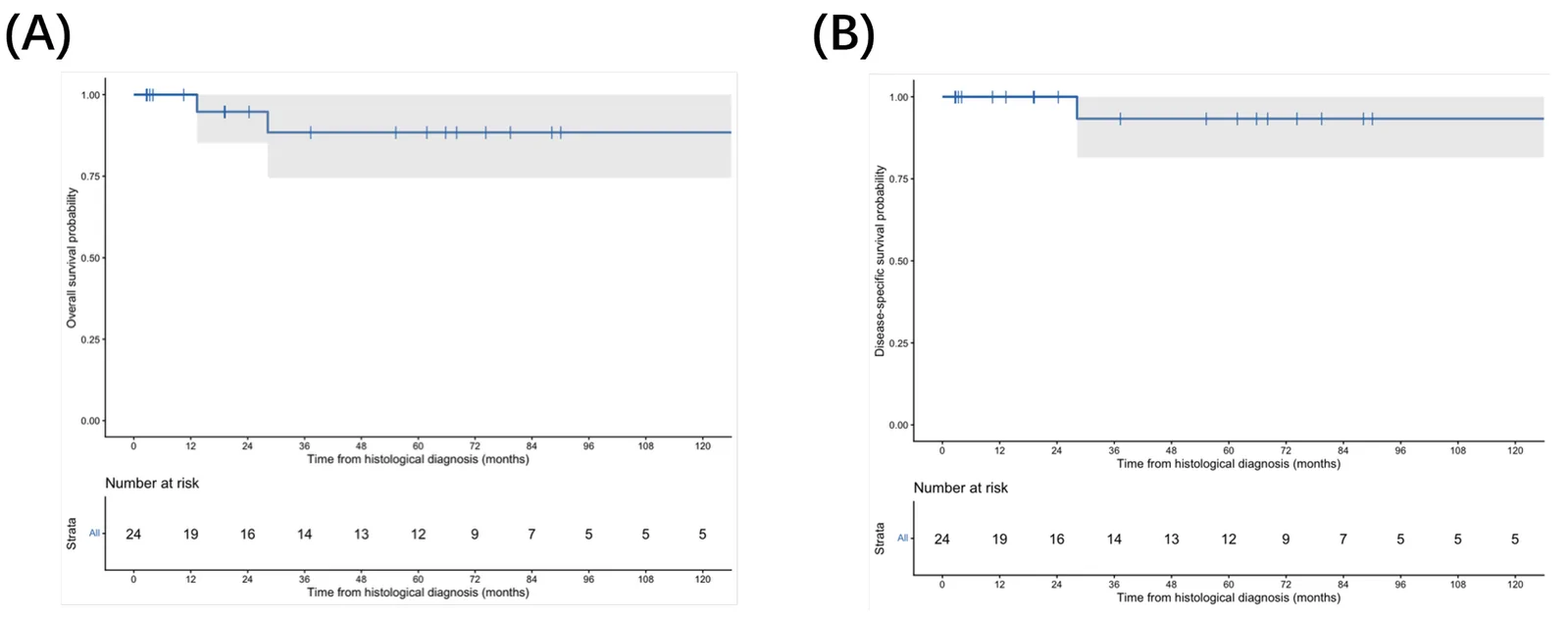
Kaplan–Meier overall survival (**A**) and disease-specific survival (**B**) curves for 24 patients with adenoid cystic carcinoma of the breast. Overall survival (OS) was defined as time from date of histological diagnosis to death from any cause or last follow-up. Disease-specific survival (DSS) was defined as time from date of histological diagnosis to death attributable to adenoid cystic carcinoma; patients who died from unrelated causes were censored at their date of death. The estimated 5- and 10-year OS were both 88.4% (95% CI 74.5–100%) and the estimated 5- and 10-year DSS were both 93.3% (95% CI 81.5–100%). Vertical tick marks indicate censored observations. Numbers at risk are shown below each curve. Shaded regions represent 95% confidence intervals. Median follow-up was 58.6 months (range 2.7–273.5 months) ACC = adenoid cystic carcinoma; DSS = disease-specific survival; OS = overall survival.

Six patients died during the study period: two from relapsed and progressive ACC, and four from unrelated causes. Of those dying from unrelated causes, two had no evidence of ACC present at the time of death, and two died with disease present. Eighteen patients (75%) were alive at last follow-up. Kaplan-Meier overall and disease-specific survival curves are presented in Fig. 2A,B, respectively, and outcome data are summarised in Table 4.

**Table 4:** Outcome summary for patients with breast ACC.

Outcome Measure	Value
**Disease relapse**	
Patients relapsed, n (%)	5 (21%)
Median relapse-free interval, years (range)	2.3 (1.3–14.0)^1^
Sites of relapse	Lung, chest wall, bone, local breast
Solid-basaloid subtype among relapsed patients	2/5 (40%)^2^
**Treatment at relapse**	
Received active treatment, n (%)	4/5 (80%)
Surgery	2 (40%)
Chemotherapy	2 (40%)
Radiotherapy	2 (40%)
No active treatment documented, n (%)	1/5 (20%)
**Survival**	
Alive at last follow-up, n (%)	18 (75%)
Total deaths, n (%)	6 (25%)
Died from ACC	2 (8%)
Died from other cause without disease	2 (8%)
Died from other cause with disease present	2 (8%)
5- and 10-year OS, % (95% CI)	88.4% (74.5–100%)
5- and 10-year DSS, % (95% CI)	93.3% (81.5–100%)
5- and 10-year RFS, % (95% CI)	82.2% (65.8–100%)

^1^Median calculated from 5 relapsed patients. ^2^Both solid-basaloid ACC patients in the cohort experienced disease relapse (2/2, 100%), compared with 3 of 22 non-SB-ACC patients (14%).

## 4 Discussion

In this retrospective 20-year single-institution analysis, we characterise the clinicopathological features and long-term outcomes of 24 patients with breast ACC treated at The Royal Marsden NHS Foundation Trust. To our knowledge, this represents the largest single-institution series of breast ACC reported to date. Our data corroborate and extend findings from prior smaller series and registry-based analyses, providing important real-world evidence to inform the management of this rare tumour.

The estimated 5- and 10-year overall survival of 88.4% (95% CI 74.5–100%) and 5- and 10-year disease-specific survival of 93.3% (95% CI 81.5–100%), despite the triple-negative immunophenotype of all tumours, underscore the fundamental biological and clinical distinction between breast ACC and other TNBC subtypes. These figures contrast sharply with the substantially worse long-term survival reported for conventional TNBC [28], and are consistent with published series reporting favourable long-term survival outcomes in breast ACC [3,7,29], confirming that the triple-negative designation carries a profoundly different prognostic implication in this histological subtype. The higher DSS relative to OS in our study reflects four deaths from unrelated causes, emphasising that mortality in this cohort was predominantly driven by competing causes rather than ACC itself. Our data provide further evidence that breast ACC constitutes a clinically and biologically distinct disease entity that must be considered separately from conventional TNBC in both clinical practice and future research frameworks.

The clinicopathological profile of our cohort reflects established features of breast ACC. The predominance of classic ACC subtype (88%), T2 tumour stage (58%), and grade 2 histology (50%) is consistent with the published literature and with the typical presentation of this malignancy as a well-circumscribed, slow-growing mass detected at an intermediate tumour size. The 33% rate of T1 disease suggests that a significant proportion of patients were diagnosed at an early, potentially screen-detected stage, underscoring the importance of radiological familiarity with ACC appearances on mammography and ultrasound [30].

The overall nodal positivity rate in our cohort was 8%, comprising one patient with sentinel node micrometastasis (N1mi) and one patient with nodal macrometastasis (N1). This is consistent with published nodal positivity rates of less than 10% in breast ACC [31,32], and our data are consistent with published evidence suggesting that extensive axillary surgery may rarely be indicated in this histological subtype. These findings raise an important clinical question regarding whether SLNB itself is necessary as a routine staging procedure for all patients with breast ACC, or whether the low pretest probability of nodal disease justifies a more selective approach. Larger multicentre studies are needed to address this question prospectively, and international consensus guidelines specific to breast ACC are warranted.

Breast-conserving surgery with adjuvant radiotherapy was the predominant treatment approach. The high rate of adjuvant radiotherapy use (88%) reflects standard practice following breast-conserving surgery and is consistent with evidence supporting locoregional radiotherapy in this disease [8,33]. Whether the absolute benefit of adjuvant radiotherapy in this low-risk subtype justifies its use universally, particularly in elderly patients or those with significant comorbidities, remains an area requiring further study. The tolerability and long-term sequelae of radiotherapy are important patient-centred considerations given the favourable overall survival expected in this patient population.

The use of systemic chemotherapy in our cohort warrants specific comment. One patient received neoadjuvant chemotherapy and achieved a response with no residual ACC identified at surgery; however, residual invasive ductal carcinoma was present, suggesting dual pathology with differential chemosensitivity of the two tumour components. This patient subsequently developed local recurrence with carcinoma exhibiting neuroendocrine features, most likely representing progression of the residual IDC component rather than ACC recurrence, and highlighting the diagnostic complexity that can arise in this setting. One further patient received adjuvant chemotherapy. These cases notwithstanding, with only two patients receiving chemotherapy for breast ACC in this cohort, no conclusions regarding chemotherapy sensitivity or resistance can be drawn. The limited use of chemotherapy likely reflects both selection bias at a specialist centre and variability in treatment approaches over the 20-year study period. Our observations are consistent with the general consensus that systemic chemotherapy offers limited benefit in early-stage breast ACC and should not be administered routinely on the basis of triple-negative receptor status alone. In the absence of prospective evidence, treatment decisions should be individualised, taking into account tumour subtype, stage, patient comorbidities, and multidisciplinary team discussion. This is particularly pertinent given the typical post-menopausal demographic of breast ACC patients, in whom chemotherapy toxicity profiles, including cardiotoxicity, peripheral neuropathy, and haematological toxicity, may be less well tolerated. Oncologists should be aware that the molecular profile of breast ACC, including its low mutational burden, absence of *TP53* mutations in classic ACC, low genomic instability, and lack of *BRCA* pathway alterations [19], differs fundamentally from that of conventional TNBC and likely underlies its relative insensitivity to platinum-based and anthracycline-based chemotherapy regimens.

A notable observation in our cohort is the markedly higher relapse rate among solid-basaloid ACC (SB-ACC) patients compared with non-SB-ACC patients (2/2, 100% vs. 3/22, 14%). While the absolute numbers are small (n = 2 SB-ACC), this finding is consistent with the emerging literature characterising SB-ACC as a biologically and clinically distinct entity with an appreciably worse prognosis than classic ACC. Shamir et al., in a series of 16 SB-ACC cases, reported a disease-related mortality of 31% with a further 19% alive with distant metastasis at the time of reporting [18]. In a multi-institutional study of 76 breast ACC cases, Khoury et al. demonstrated that basaloid morphology was the only independent predictor of recurrence-free survival on multivariate analysis (HR 3.87, *p* = 0.038) [34]. Taken together with our observation that both SB-ACC patients experienced relapse compared with 14% of non-SB-ACC patients, these data from independent series consistently support the classification of SB-ACC as a biologically aggressive variant warranting separate clinical consideration. While responses to neoadjuvant chemotherapy are generally poor in SB-ACC, with no pathological complete responses observed among seven treated patients in one series, [18] isolated cases of near-complete pathological response to platinum and anthracycline-based regimens have been reported [35], suggesting that chemotherapy should not be categorically withheld in the locally advanced or high-risk setting. These data suggest that SB-ACC may warrant management approaches more akin to conventional TNBC than to classic ACC in selected patients, though this requires validation in larger prospective series. This highlights the importance of accurate WHO subtype classification at the time of diagnosis.

The median relapse-free interval of 2.3 years (range 1.3–14.0 years) and the pattern of relapse observed in our cohort have important implications for surveillance strategies. Notably, the estimated 5-year and 10-year RFS were identical at 82.2% (95% CI 65.8–100%), with no relapses observed between 5 and 10 years of follow-up. Two further relapses occurred beyond 11 years, at 11.3 and 14.0 years respectively. One patient in our cohort survived 23 years from initial diagnosis before succumbing to progressive ACC, illustrating the extraordinarily protracted natural history that can be observed in this malignancy. Unlike conventional TNBC, where recurrence risk is front-loaded in the first 1–3 years after diagnosis, breast ACC can recur many years or decades after initial treatment [27]. These observations support the case for long-term follow-up beyond the standard 5-year surveillance window, though optimal surveillance strategies have not been prospectively evaluated.

The *BRCA* germline testing data in our cohort, while incomplete due to the retrospective design and evolving clinical practice over the study period, are informative. Of the seven patients with available results, all were *BRCA* wild type, consistent with the molecular characterisation of breast ACC as a genomically distinct entity from *BRCA1*-associated basal-like TNBC. Clinicians should be aware that the triple-negative phenotype of breast ACC does not carry the same probability of underlying germline *BRCA* pathogenic variants as conventional TNBC.

Limitations of this study include its retrospective design and the small sample size inherent to the rarity of breast ACC. The small cohort size limits the statistical power of all analyses and findings should be considered hypothesis-generating rather than practice-defining. Ki-67 proliferative index data were unavailable in 79% of patients, reflecting inconsistent documentation over the 20-year study period and the evolving clinical recognition of Ki-67 as a prognostically relevant biomarker in breast cancer. This substantially limits the ability to characterise the proliferative biology of this cohort, and the available Ki-67 data in this series should be interpreted with caution given the small number of patients with documented values (n = 5, 21%). *BRCA* germline status was similarly unavailable in 71% of patients, reflecting evolving clinical practice regarding germline testing over the study period. No molecular profiling was performed in this cohort, and the molecular discussion draws entirely on published literature to provide biological context for the clinical observations reported rather than describing molecular data generated in the present study. Prospective molecular characterisation of breast ACC, including *MYB-NFIB* fusion status, tumour mutational burden, and *BRCA* pathway analysis, would substantially strengthen future studies in this area. The formal three-subtype WHO classification of breast ACC was introduced in the 5th edition (2019). For patients diagnosed prior to this, subtype classification was recorded as documented in the clinical pathology report at the time of diagnosis. The possibility of reclassification under contemporary WHO criteria cannot therefore be excluded, particularly for cases diagnosed in the earlier part of the study period, and this should be acknowledged when interpreting subtype-specific findings. Treatment variability over the 20-year study period is a further limitation, as management approaches evolved considerably during this time. Survival estimates at extended timepoints are based on small numbers of patients at risk, resulting in wide confidence intervals, and should be interpreted accordingly. No comparative statistical analyses between WHO subtypes were performed given the small subgroup sizes and subtype-specific findings are presented descriptively and should be considered hypothesis-generating. Prospective validation in larger multicentre cohorts is warranted to confirm these findings and expand the evidence base for this rare malignancy.

## 5 Conclusions

This single-institution cohort study, contributes to the limited evidence base for this rare malignancy by providing detailed clinicopathological characterisation, WHO subtype distribution, long-term survival estimates extending beyond 20 years, and real-world management data from a high-volume specialist National Health Service centre. The estimated 5- and 10-year overall survival of 88.4% (95% CI 74.5–100%) and disease-specific survival of 93.3% (95% CI 81.5–100%) are consistent with the largest published comparators and contrast sharply with the long-term outcomes reported for conventional TNBC [28] confirming the fundamentally distinct prognosis of breast ACC despite its triple-negative immunophenotype. The clinical behaviour of breast ACC more closely resembles salivary gland ACC than conventional triple-negative breast cancer, characterised by low rates of nodal involvement, limited chemotherapy sensitivity, and excellent locoregional control with surgery and radiotherapy. The estimated 5- and 10-year relapse-free survival of 82.2% (95% CI 65.8–100%), with no relapses between 5 and 10 years but two beyond 11 years and one patient surviving 22.8 years before succumbing to progressive ACC, supports the case for long-term surveillance extending well beyond the standard 5-year window. The markedly higher relapse rate among solid-basaloid ACC patients (100% vs. 14% for non-SB-ACC patients) highlights the importance of accurate WHO subtype classification and suggests that SB-ACC may warrant a more intensive management approach in selected patients, pending validation in larger multicentre cohorts. These findings suggest that a conservative, locoregionally focused management approach may be appropriate for classic ACC in selected patients and raise questions about the routine use of adjuvant chemotherapy solely on the basis of triple-negative receptor status. Multicentre data and molecular studies are needed to further define optimal management strategies across all disease settings, from early-stage locoregional treatment to systemic therapy in the advanced and metastatic setting.

## Data Availability

The data presented in this study are not publicly available due to institutional governance restrictions and the inclusion of potentially identifiable patient information. De-identified data may be made available from the corresponding author upon reasonable request and subject to institutional approval and data sharing agreements, in accordance with applicable data protection regulations.

## Abbreviations

The following abbreviations are used in this manuscript:

Abbreviation	Definition
ACC	Adenoid cystic carcinoma
ALND	Axillary lymph node dissection
DSS	Disease-specific survival
EHR	Electronic health records
ER	Oestrogen receptor
HER2	Human epidermal growth factor receptor 2
HGT-ACC	Adenoid cystic carcinoma with high-grade transformation
IDC	Invasive ductal carcinoma
Ki-67	Proliferative index marker
KM	Kaplan-Meier
LVI	Lymphovascular invasion
N1mi	Nodal micrometastasis
NHS	National Health Service
OS	Overall survival
OSNA	One-step nucleic acid amplification
PR	Progesterone receptor
RFS	Relapse-free survival
SB-ACC	Solid-basaloid adenoid cystic carcinoma
SLNB	Sentinel lymph node biopsy
TNBC	Triple-negative breast cancer
WHO	World Health Organisation
WLE	Wide local excision
